# Identification of New DR5 Agonistic Nanobodies and Generation of Multivalent Nanobody Constructs for Cancer Treatment

**DOI:** 10.3390/ijms20194818

**Published:** 2019-09-27

**Authors:** Golnaz Sadeghnezhad, Ema Romão, Robert Bernedo-Navarro, Sam Massa, Khosro Khajeh, Serge Muyldermans, Sadegh Hassania

**Affiliations:** 1 Faculty of Biological Sciences, Tarbiat Modares University, Teheran 14115-331, Iran; golnaz.sadeghnezhad@gmail.com (G.S.); khajeh@modares.ac.ir (K.K.); 2 Cellular and Molecular Immunology, Vrije Universiteit Brussel, Brussels 1050, Belgium; ema.estevens.romao@vub.be (E.R.); sam.massa@vub.be (S.M.); 3 Laboratory of Environmental and Life Sciences, University of Nova Gorica, Nova Gorica 5000, Slovenia; robert.bernedo@ung.si

**Keywords:** death receptor 5, nanobody, TRAIL, cancer therapy, apoptosis

## Abstract

Current cancer therapeutics suffer from a lack of specificity in targeting tumor cells and cause severe side effects. Therefore, the design of highly specialized drugs comprising antibody derivatives inducing apoptosis in targeted cancer cells is considered to be a promising strategy. Drugs acting on death receptor 5 (DR5) such as DR5 agonist antibodies replacing “TNF-related apoptosis-inducing ligand” (TRAIL) offer feasible opportunities in this direction. Although such agonists provided good antitumor activity in preclinical studies, they were less effective in clinical studies, possibly due to a disturbed Fc interaction with Fc-γ receptors. Thus, multimerized antigen binding fragments without Fc have been proposed to increase their efficacy. We generated nanobodies (Nbs), recombinant variable domains of heavy chain-only antibodies of camelids, against the DR5 ectodomain. Nb24 and Nb28 had an affinity in the nM and sub-nM range, but only Nb28 competes with TRAIL for binding to DR5. Bivalent, trivalent, and tetravalent constructs were generated, as well as an innovative pentameric Nb complex, to provoke avidity effects. In our cellular assays, these trimeric, tetrameric, and pentameric Nbs have a higher apoptotic capacity than monomeric Nbs and seem to mimic the activity of the natural TRAIL ligand on various cancer cells.

## 1. Introduction

Cancer ranks second among the common primary causes of death worldwide, and it is anticipated to have accounted for 9.6 million deaths in 2018 [1]. The design of potent targeted therapies involving monoclonal antibodies to target specifically cancer cells constitute a promising strategy to treat tumors in cancer patients [2]. 

“Evading apoptosis” is an important hallmark of cancer progression and most apoptotic programs divide into the intrinsic or the extrinsic pathway. The extrinsic pathway of apoptosis is triggered by signals that employ extracellular death receptors (DR), and the manipulation of these receptor activation mechanisms has been considered as a tempting therapeutic strategy to induce, exclusively, apoptosis in tumor cells [3]. Among DRs, death receptor 5 (DR5), also known as TRAIL-R2 or KILLER receptor, is the most promising candidate to develop a targeted therapy against cancer, since its expression level is significantly higher in cancer cells compared to that of healthy cells. Therefore, its triggering may potentially mediate selective activation of apoptosis in cancer cells and thus their killing [4].

DR5 is a type I transmembrane protein consisting of three regions: an extracellular, a transmembrane and an intracellular part. The last part comprises a homologous cytoplasmic sequence of the death domain for interaction with Fas associated death domain (FADD) and other members of the apoptosis signaling pathway. The extracellular region has 2–4 concatenated cysteine-rich domains (CRDs) that are responsible for ligand binding [5]. The N-terminal CRD1 of DR5 forms a pre-ligand association domain (PLAD), while a GXXXG motif in the transmembrane helix (TMH) harbors the interaction sites for the dimeric state of inactive DR5 that inhibits apoptosis signaling in absence of TNF-related apoptosis-inducing ligand (TRAIL) [6]. 

TRAIL is an important actor within the apoptosis scenario. It is a 281–amino acid type II transmembrane protein that belongs to the Tumor Necrosis Factor (TNF) superfamily, targets death receptors (DR5 and DR4), membrane attached decoy receptors (DcR1 and DcR2), and also a soluble decoy receptor (Osteoprotegrin). These three decoy receptors compete with the death receptors for TRAIL binding and are thus blocking the apoptotic signals [7]. The extracellular part of membrane-attached TRAIL can be shed after limited proteolysis by cysteine proteases. The self-assembling TRAIL protein forms a noncovalent–bonded homo-trimer with a pyramid-like architecture. Each monomer contains one single cysteine (Cys-230), which is involved in the chelation of a zinc ion in the corner of the pyramid, that is requisite for the native structure, stability and, thus, the biological activity of TRAIL [8]. TRAIL also contains a significant number of aromatic amino acid residues, of which eight provide a hydrophobic surface interacting with neighboring subunits and stabilizing the inner core of the trimer [5,8].

To date, various strategies have been developed to target TRAIL receptors. An ideal therapeutic agent to activate TRAIL-dependent apoptosis is supposed to have an affinity that is comparable to that of the natural ligand, (i.e., single digit nM affinity or lower), and a prolonged circulation time in the bloodstream. Several drawbacks are encountered with the use of recombinant human TRAIL, such as a specificity for its different types of receptors and therefore leading to various and unpredictable functions in the body, short elimination half-life, and in some cases hepatotoxic effects. Consequently, better alternatives are required, such as DR5 agonistic antibodies capable of interacting only with death receptors. Such antibodies are easy to produce, relatively safe to use, exhibiting improved pharmacokinetic properties compared to recombinant TRAIL, and are highly specific for only one single type of receptor [4]. 

Although such agonists demonstrate significant anti-tumor activity in preclinical models, the clinical efficacy in human cancer patients has been disappointingly low. One possible explanation might be that the current classes of therapeutic molecules are insufficiently robust to elicit a significant response in patients. In particular, the dimeric antibody agonists require a secondary cross-linking via Fcγ receptors expressed on immune cells to achieve an optimal clustering of DR5 [9]. Thus, the need to develop better receptor aggregating and clustering molecules is large, and this stimulated us to design multivalent Nanobodies (Nbs) aiming to produce a significantly stronger DR5 agonist [10]. A Nb is a recombinant single domain antigen-binding fragment derived from heavy chain–only antibodies circulating in blood of Camelidae [11]. Nbs are easy to generate, and they bind their cognate antigen with high specificity and affinity [12]. They have a good biodistribution and a fast tissue penetration. Nbs are robust and easy to engineer into multimeric constructs, and the first Nb-based construct has recently been FDA approved [13]. 

The objectives of the present study are manifold: (i) identify high affinity DR5-specific Nbs (ii) that recognize a TRAIL-overlapping-epitope on DR5, (iii) unveil the possible correlation of an increasing valency of Nbs in the cell killing activity, and, finally, (iv) demonstrate that DR5-specific Nbs activate apoptosis in target cells. Overall, we demonstrated that our trivalent DR5-targeting Nbs mimic the activity of the natural TRAIL ligand. Moreover, increasing the valency of the nanobody domains to a tetrameric or pentameric constructs (assisted by pentameric Shiga-like toxin 2a B domain (STx2aB_5_) [14] markedly increased the cell-killing potency on various tumor cell lines. The increased potency was attributed to the faster kinetics of assembling the death-inducing signaling complex and caspase 3/7 activation. 

## 2. Results

### 2.1. Production of Recombinant DR5 and TRAIL in E. coli SHuffle B 

**Figure 1:**
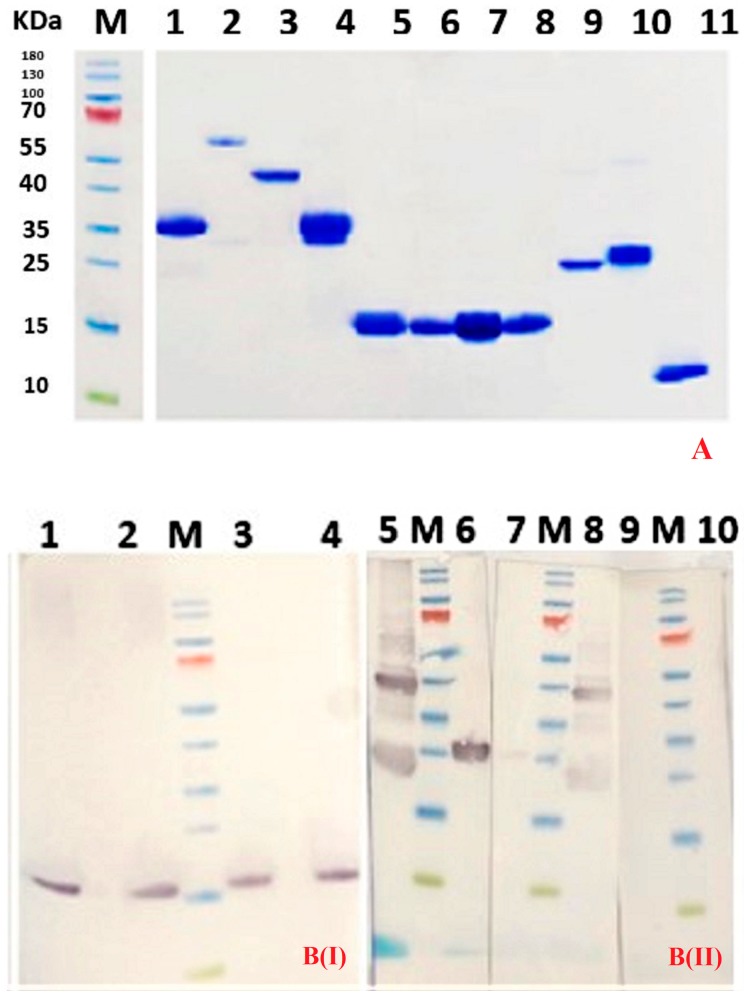
**Panel A:** SDS-PAGE gel stained with Coomassie blue R250. Lanes from left to right: lane M: protein size marker with the molecular mass indicated in KDa, Lane 1: Nb28-113, lane 2: Nb28_4_; lane 3: Nb28_3_; lane 4: Nb28_2_; lane 5: Nb24; lane 6: Nb22; lane 7: Nb28; lane 8: Nb50, lane 9: TRAIL; lane 10: DR5; lane 11: Stx2a-B_5_. All samples were treated with dithiotreitol before electrophoresis. **Panel B**(**I**) Western blot analysis of different Nbs used in this study: Lane 1: Nb22; lane 2: Nb24; lane 3: Nb28; lane 4: Nb50. The membrane was treated with anti HA tag monoclonal antibody and goat anti mouse IgG conjugate. Lane M contains protein size markers. **Panel B** (**II**) Western blot analysis revealing the interaction between DR5 and Nb24 or Nb28. Lanes 5 and 6: DR5 without or with dithiotreitol and probed with Nb24; Lanes 7,8: DR5 with or without dithiotreitol and probed with Nb28. Lanes 9 and 10: DR5 without or with dithiotreitol (membranes were not incubated with Nb). All membranes were incubated with anti HA tag monoclonal antibody and goat anti mouse IgG conjugate. Lanes M contain protein size markers.

The expression plasmid vectors pET-28a/trail or pET-28a/dr5 were transformed into SHuffle B cells for production of soluble TRAIL and ectodomain DR5 proteins, respectively. These cells manage to correctly fold disulfide bonded proteins in their cytoplasm, mediated by Gor and TrxB reductases, along with DsbC disulfide bond isomerase [15]. The cells were cultured in the presence of 1 mM IPTG, and the cytoplasmic target protein was purified from the cell lysate using IMAC on Ni^2+^-NTA resin and SEC on Superdex-75 equilibrated with PBS (TRAIL) or acetate (DR5) buffer. The purified proteins were electrophorized under reducing conditions on 12% polyacrylamide gel. The Coomassie stained proteins demonstrated the homogeneity and purity of TRAIL and DR5 (Figure 1). 

The crystallized extracellular domain of DR5 shows seven intra-disulfide bonds [16,17,18]. However, the DR5 ectodomain produced in SHuffle B cells most likely formed also inter-disulfide bonds, as can be inferred from the band with molecular weight (MW) around 40,000 that is only visible when the sample is run on gel under non-reducing conditions (Figure 1B, panel II, lanes 5 and 8). The dimerized material noticed on gel when run under non-reducing conditions possibly reflects the Pre-Ligand Assembly Domain (PLAD).

### 2.2. Selection of DR5-Binding Nbs

After immunizing a dromedary with the DR5 ectodomain, the nanobody repertoire of the heavy chain–only antibodies was cloned according to standard protocols, and eight different nanobodies with specificity toward the ectodomain of DR5 were retrieved after phage display [19]. The specificity of these Nbs was confirmed in a monoclonal phage ELISA. The gene of the four most promising Nbs was recloned in bacterial expression vector pMECS and transformed in WK6 cells, for periplasmic production with a C-terminal haemagglutinin (HA) and a hexahistidine (His_6_) tag. Of these Nbs, referred to as Nb22, Nb24, Nb28, and Nb50, Nb24 and Nb50 possess a VH imprint in their framework region 2 (FR2) (Val42, Gly49, Pro50, and Trp52), whereas Nb28 and Nb22 harbor the FR2 amino acids characteristic for a VHH (Phe42, Glu49, Arg50, and Gly52). The MW, isoelectric point and extinction coefficient for all nanobodies were calculated in silico using the ExPasy ProtParam online platform. The recombinant Nbs were purified from periplasmic extracts of WK6 cells, by IMAC and SEC. The purified proteins were analyzed by SDS-PAGE and stained with Coomassie Blue R-250 (Figure 1A). The apparent MW of each Nb is close to 15,000 as expected from the MW calculations, and the same bands were also revealed after western blot using anti-His or anti hemagglutinin antibodies as probe (Figure 1B, panel I). Since the yield of purified Nb22 (0.5 mg Nb/L of culture) and Nb50 (0.66 mg/L) was much lower than that of Nb24 (1.2 mg/L) and Nb28 (2.6 mg/L), we selected the latter two Nbs for further investigations. 

### 2.3. Specificity of Nbs 

The specificity of the Nbs for DR5 was assessed via western blot and fluorescent activating cell sorting (FACS). For western blot, the recombinant DR5 ectodomain was first separated on an SDS-polyacrylamide gel under reducing and non-reducing conditions. Since Nbs prefer conformational epitopes [12], they usually perform poorly as a probe in western blots, certainly if the target protein has been reduced before electrophoresis. Remarkably, our two selected nanobodies recognize very well the DR5 separated on a gel under non-reducing conditions (Figure 1B, panel II). Specifically, the dimerized DR5 fraction is very well recognized by both Nb24 and Nb28 in western blot. Treating the DR5 ectodomain with a reducing agent (e.g., dithiotreitol) before electrophoresis revealed one single intense band in western blot when probed with Nb24. In contrast, the reduced DR5 protein could barely be detected with Nb28 in western blot (Figure 1B, panel II). This result may indicate that Nb24 recognizes a linear epitope, while Nb28 prefers a conformational epitope. 

**Figure 2:**
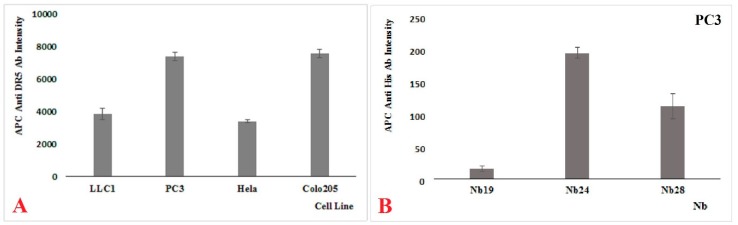
(**A**) Fluorescent activating cell sorting (FACS) data showing the expression of DR5 on various cell lines as probed with commercial DR5 specific antibodies. (**B**) Recognition of antigens on PC3 cell by DR5-specifc Nb24 and Nb28. Nb19 is specific for a bacterial adhesin and is used as negative control. The standard errors of the mean are obtained as the standard deviation divided by the root of the size of the sample (*n* = 3)

PC3, HeLa, and Colo205 are human cancer cell lines that were shown in FACS to express DR5 on their surface when probed with commercial antibodies against human DR5. The signal intensity for PC3 and Colo205 is almost identical, whereas that for HeLa cells was significantly lower (Figure 2A), probably because they express less DR5 on their membrane. The LLC1 is a mouse lung cancer cell line, and it is well established that mice possess one death receptor (mDR5) that is homologous to human DR5 and two decoy receptors (mDcR1 and mDcR2) for TRAIL [20]. Our FACS result with the hDR5-specific commercial antibody indicates that this monoclonal cross reacts with mDR5 and it confirms that mouse LLC1 cells express mDR5 on their surface as the signal intensity is comparable to that of the HeLa cells (Figure 2A). 

Since the semi adhesive Colo205 cell line is more difficult to handle and since the shape and appearance of PC3 cells is more suitable for monitoring cell death, we prefer to focus primarily on the PC3 cell line to assess the recognition of its DR5 by our Nbs (Figure 2B). The attachment of Nb24 and Nb28 (both carrying an His_6_ tag) on the PC3 cells is evident after staining with anti-His_6_ antibodies, in contrast to the clear absence of Nb19 interaction with PC3 (Nb19 recognizes a bacterial adhesin). Of both Nbs, Nb28 has the higher affinity for DR5 (see further). Nevertheless, Nb24 gives a higher signal intensity in FACS, probably because it has a better access to DR5 when surface exposed on the membrane of PC3 cells. 

### 2.4. Affinity Measurement of Nbs to DR5

Next, we decided to measure the kinetic binding parameters of the Nb–DR5 interaction via surface plasmon resonance (SPR). The purified recombinant human DR5, eluting from SEC and containing a significant fraction of dimerized material, was immobilized on a CM5 sensor chip. Different concentrations of monomeric and multivalent anti-DR5 nanobodies in HBS (10 mM HEPES, 3.4 mM EDTA, 150 mM NaCl, 0.005% Tween-20) were flown over the sensor chips in Biacore T200 for the kinetic binding analysis. The kinetic on and off rates were determined from the sensorgrams and used to calculate the equilibrium dissociation constant (K_D_) (Table 1). Apparently, Nb24 and Nb28 exhibited a low K_D_ value in the single digit nM and sub-nM range, respectively. The binding kinetics and K_D_ of the monomeric Nb28 are very close to those of the recombinant trimeric TRAIL (Table 1).

We also generated bivalent, biparatopic, bispecific, and multivalent Nb constructs using the wide-spread (Gly_4_Ser)_3_ linker [21,22,23], selected for its length and flexibility so that each Nb retains sufficient rotational freedom and providing a spacing of approximately 50 Å between subunits when stretched. A first type of multivalent Nb comprised a tandem repeat of the same (e.g., Nb28_2_) or different Nbs (e.g., Nb24-28 or Nb28-24) to generate bivalent or biparatopic constructs, respectively. Another type of construct consists of the DR5-specific Nb24 or Nb28 fused with Nb113 directed against serum albumin [14], here referred to as Nb24-A and Nb28-A, respectively.

In comparison to the monovalent Nb28, the k_on_ of bivalent Nb28_2_ for DR5 remained similar, whereas its k_off_ improved more than 10-fold so that the apparent affinity increased 13-fold, due to avidity effects (Table 1). Converting the Nb28 to a trivalent construct (Nb28_3_) did not show any further affinity improvement. 

The Rmax value obtained after flowing saturating amounts of the monomeric Nb28 (~117 RU) increases to exactly the double Rmax value for the Nb28-A (~234 RU). This is expected if each DR5 molecule from both, the monomeric DR5 fraction and from the dimerized DR5 fraction, is bound by one Nb28 moiety either from the monomer Nb28 with molecular mass of 15 kDa or from the bispecific Nb28-A with molecular mass of 30 kDa. Interestingly, the Rmax values of the bivalent Nb28_2_ (~145 RU) and the trivalent Nb28_3_ (~154 RU) increased to lower levels than those for the Nb28-A bispecific construct, which indicates that both Nb28 nanobodies within the bivalent construct are binding simultaneously to the two DR5 molecules within the dimerized DR5 molecules. Furthermore, it also shows that both molecules within the DR5-dimer capture only one single Nb28_2_ or Nb28_3_ molecule instead of two molecules.

The binding parameters of the biparatopic constructs, Nb28-Nb24 and Nb24-Nb28, were not significantly different from the bivalent or trivalent Nb28. However, the Nb24-28 exhibited a slightly faster k_off_ value, indicating that Nb28 when positioned at the C-end of the construct is slightly less effective in antigen recognition compared to its N-terminal location, possibly due to interference from the upstream linker. In addition, the Rmax values (~180–185 RU) for both biparatopic constructs, which is approximately halfway between the values for the bivalent Nb28_2_ and the Nb28-A or Nb24-A, show that a considerable fraction of the biparatopic construct has its Nb28 and Nb24 molecule associated with one DR5 molecule even if present as DR5-dimer. 

### 2.5. Epitope Binning

**Figure 3:**
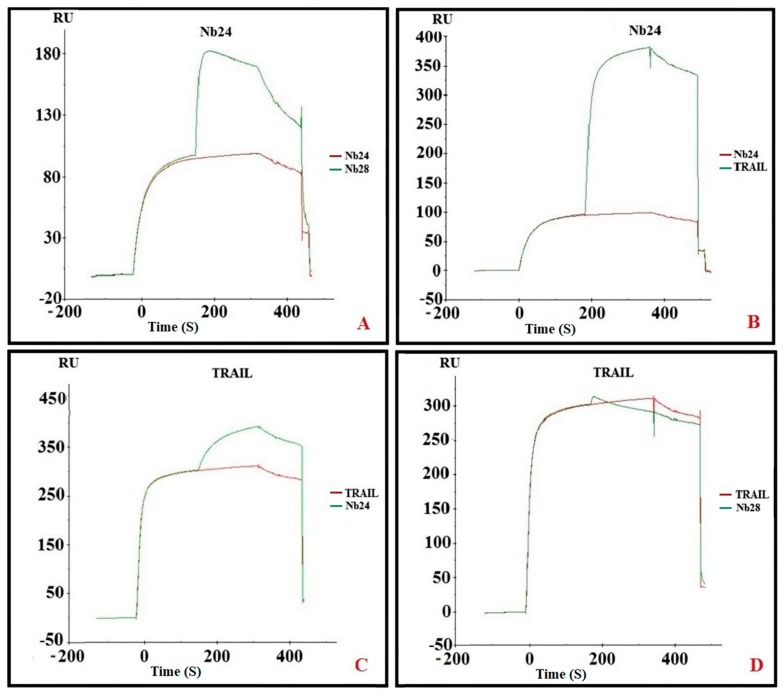
Sensorgrams used for epitope binning. (**A**) SPR sensorgram where DR5 is first saturated with Nb24 followed by Nb24 (red curve) or a mixture of Nb24 and Nb28 (green curve). (**B**) SPR sensorgram where DR5 is first saturated with Nb24 and subsequently with Nb24 (red curve) or a mixture of Nb24 plus tumor necrosis factor–related apoptosis-inducing ligand (TRAIL). (**C**,**D**) SPR sensorgram where DR5 is first saturated with TRAIL and thereafter with TRAIL alone (red curves) or with a mixture of TRAIL and Nb24 (green curve in C) or TRAIL and Nb28 (green curve in D).

The SPR sensorgrams offer an elegant solution for epitope binning. By first saturating the ligand binding sites on the DR5 target with one Nb and then adding a mixture of the same Nb with another Nb it is possible to infer whether those Nbs compete for an overlapping epitope. This kind of analysis can assess the possible competition between TRAIL and Nbs for DR5 binding, as well. Such experiments revealed that Nb24 and Nb28 are associating at different epitopes on the DR5 molecule. Indeed, saturating DR5 with Nb24 generates a signal of around 90 RU (resonance units), and the further addition of Nb28 increases this signal to about 180 RU (Figure 3A). Likewise, if Nb24 molecules are bound to the DR5 prior to the challenge with TRAIL molecules, then TRAIL will attach to DR5::Nb24 complexes without restrictions (Figure 3B). The SPR signal of 90 RU for Nb and 280 RU for TRIAL matches the difference in molecular mass: 15,000 and 50,000, respectively. The other way around, first adding TRAIL and subsequently Nb24, confirms that both molecules bind independently from each other on DR5 (Figure 3C). In contrast, the prior binding of TRAIL molecules to DR5 seriously hampered the subsequent Nb28 binding (Figure 3D). This exercise performed with all possible Nb pairs and also Nbs and TRAIL confirmed that Nb28 and TRAIL are competing for an overlapping epitope, but Nb 24 and TRAIL (or Nb28) recognize independent epitopes on DR5. 

### 2.6. Increasing Valency of Nb28 Correlates with Cancer Cell–Killing Potency 

**Figure 4:**
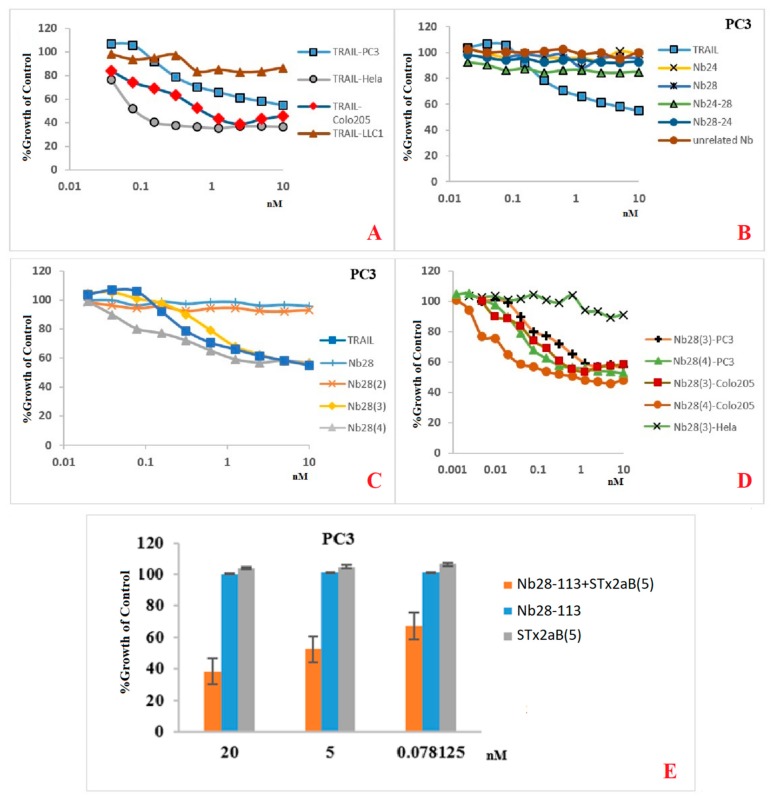
Survival of several cancer cell lines upon challenge with TRAIL or Nb constructs. (**A**) human cancer cell lines Colo205, HeLa, or PC3 or mouse cancer cell line LLC1 were exposed to human TRAIL at various concentrations and the % growth of non-treated control was measured. (**B**) The effect of TRAIL or monovalent Nb24 or Nb28, or biparatopic Nb28-24 or Nb24-28 at different concentrations on PC3 cells was followed. (**C**) The % growth of control for PC3 cells when exposed to various concentrations of TRAIL, or monovalent, bivalent or trivalent Nb28. (**D**) The % of growth of cancer cells relative to control cells was measured for PC3, Colo205, or HeLa cell when exposed to various concentrations of trivalent or tetravalent Nb28. (**E**) The growth of PC3 cells challenged with Stx2aB pentamer, with Nb28-113 or the mixture of both at three different concentrations.

We first assessed the sensitivity of three different human cancer cell lines and one mouse cancer cell line upon challenge with TRAIL. The viability of these cancer cell lines was monitored with Alarmar Blue (Figure 4A). Apparently, the growth of the mouse cancer cell line was only slightly affected by exposure to 0.03 to 10 nM TRAIL. In contrast, the growth of the human cancer cell lines in presence of TRAIL was reduced in a dose dependent manner. Growth of HeLa cells was already significantly compromised with TRAIL levels as low as 0.06 nM, whereas Colo205 cell growth was only inhibited above 0.1 nM TRAIL. A TRAIL concentration of 10 nM had a growth limitation to about 20 to 55% of the normal growth of various human cell lines. 

We then compared the possibility of our Nbs to mimic the TRAIL effect on PC3 cancer cells. The monomeric Nb24 or Nb28, the biparatopic constructs Nb24-Nb28 or Nb28-Nb24 at concentrations of up to 10 nM failed to show any significant effect on cell growth (Figure 4B). Also, the bivalent Nb28_2_ failed to inhibit the growth of these cancer cells (Figure 4C). In contrast, a trimeric Nb28_3_ and tetrameric Nb28_4_ exhibited significant growth retardation on PC3 that was similar to that of TRAIL over the same concentration range. We further tested the activity of the trivalent Nb28_3_ and tetravalent Nb28_4_ to induce cell death on various other cell lines (Figure 4D). Although the difference in effect on cell growth between Nb28_3_ and Nb28_4_ is low, the tetravalent Nb28_4_ consistently performed slightly better than the trivalent Nb28_3_ in retarding the growth of PC3 and Colo205 (Figure 4D) (and Jurkat cells, not shown). The effect on HeLa cells induced by oligomeric Nb28 was minimal (only Nb28_3_ is shown, Figure 4D). This might be explained by the reduced number of DR5 receptors on the HeLa cells compared to PC3 or Colo205 cells as scored by a commercial anti-DR5 monoclonal antibody (Figure 2A).

We wanted to investigate the performance of a pentavalent construct of Nb28 in killing cancer cells, relative to the killing induced by our Nb28_4_. However, the production yield is gradually decreasing in going from monomeric, dimeric, trimeric to tetrameric Nb constructs. We therefore evaluated another strategy: by making a bispecific Nb with Nb28 and Nb113 with specificity for the B domain of Stx2a, that is spontaneously forming stable pentamers, we could generate a pentavalent anti DR5 construct by mixing Stx2a-B_5_ and Nb28-113. Whereas the Stx2a-B_5_ or the Nb28-113 alone has no effect on the growth of PC3 cells, the mixture of both components becomes a very potent growth inhibitor (Figure 4E). 

### 2.7. Triggering DR5 with Nbs or TRAIL Elicits Caspase 3/7

A likely mechanism for the cell growth inhibition achieved by oligomeric DR5-targeting Nbs could be through the induction of the apoptotic signaling pathway. Thus, we explored the activation of the caspase 3/7 monitored via an enzymatic readout [24] after adding oligomeric Nbs to the cell cultures. In the first set of experiments, the caspase 3/7 activity in Colo205 cancer cells treated with trimeric Nb28_3_, tetrameric Nb28_4_ and bispecific Nb28-113 mixed with Stx2aB_5_ pentamer were compared to the protease activity of these cancer cells after treatment with Apo2L/TRAIL. 

**Figure 5:**
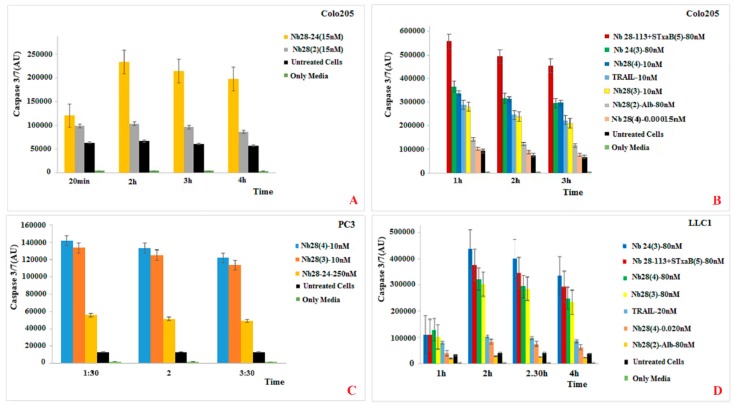
Multimeric Nb constructs or TRAIL were assessed to elicit caspase 3/7 on different cancer cells lines. (**A**,**B**) Colo205 cells were exposed to various Nb constructs or TRAIL at different concentrations as indicated in the legends. (**C**) The effect of selected biparatopic Nb24-Nb28, trivalent Nb28 or tetravalent Nb28 on PC3 cells for inducing caspase 3/7 activity. (**D**) Caspase 3/7 generation by mouse cell line LLC1 after exposure to various Nb constructs as indicated in the legend. “Untreated cells” stands for the control wells where cells were not exposed to TRAIL or Nbs. “Media” stands for the cells that were not treated with TRIAL or Nb nor chemicals to measure the caspase activity.

First, for Colo205 incubated in presence of 15 nM bivalent Nb28_2_ shows a weak induction of caspase 3/7 and a nearly twofold higher response for the biparatopic Nb Nb24-28 construct (Figure 5A). More importantly, these cells in presence of 10 nM Nb28_3_ generated a strong caspase 3/7 activity that is nearly identical to that of TRAIL at the same concentration (Figure 5B). Remarkably, the protease activity induced by tetrameric Nb28_4_ at 10 nM was even slightly higher than that of TRAIL and equivalent to that of 80 nM Nb24_3_ (of note, a binder that does not compete for TRAIL epitope). However, the presence of 0.15 pM Nb28_4_ or 80 nM Nb28_2_-A failed to induce any caspase 3/7 activity above background. Furthermore, the pentavalent construct turned out to be the best caspase 3/7 inducer. This demonstrates the specificity of the Nb28 from the trivalent construct onward and the absence of contaminating effector molecules in our recombinant protein preparations. Interestingly, the caspase 3/7 activity lasted for several hours.

The same picture emerges from experiments with human PC3 cells (Figure 5C). Strong caspase 3/7 induction is observed when cells are incubated with 10 nM trivalent or tetravalent Nb28, whereas the biparatopic Nb construct generates less than half the signal, even at 25-fold higher concentrations. 

Finally, we tested the responsiveness of the mouse cell line LLC1 for TRAIL and the Nb constructs (Figure 5D). A bivalent construct Nb28_2_ joined to the serum albumin binding Nb-SA1 used at 80 nM provoked no signal above background. However, the same molar concentration of trivalent Nb24_3_ or Nb28_4_, or tetravalent Nb28_4_ or Nb28-113 mixed with Stx2aB_5_ released a high level of caspase 3/7 activity. This signal is much higher than that obtained for 20 nM human TRAIL on this mouse cell line. 

## 3. Discussion

In view of the importance of exploring new anticancer agents we aimed to identify agonistic Nbs against DR5, a crucial target to transduce apoptotic signaling in cancer cells [25,26,27].

In a previous effort, we generated Nbs against this target [19], and in this report, we focus on two of these Nbs. Both Nbs recognize recombinant DR5 as shown in western blot, and they also recognize Colo205, PC3, and HeLa cells to various degrees in FACS, suggesting that they might recognize the native protein on human cancer cell lines as well. Also, mouse cancer cell line LLC1 was shown to react with our Nbs, indicating a cross reactivity between the mouse and human target. However, our Nbs differed from each other in many ways. Nb24 has a “VH-like” imprint in its FR2 and associates with nM affinity, most probably to a linear epitope on DR5 that is outside the binding site of TRAIL. In contrast, Nb28 has a clear VHH imprint in its FR2 and binds with sub-nM affinity to a conformational epitope that overlaps with that of TRAIL. According to our SPR measurements, the recombinant trimeric TRAIL protein also recognizes the DR5 (monomer and dimer mixture) with an apparent affinity of around 0.5 nM (Table 1). Interestingly, the monomeric, monovalent Nb28 had nearly identical k_on_ and k_off_ rates than the trimeric TRAIL on our DR5 preparations. 

Of note, the interaction of Nbs with DR5 and competition with TRAIL was performed with recombinant proteins. Nbs normally are easy to produce recombinantly in *E. coli*. Although oligomeric Nbs could still be expressed in shake flasks and purified by IMAC and SEC in mg levels per L of culture, we noticed that the expression lowered with each extra Nb within the repeats. Especially oligomeric constructs with Nb24 were sometimes very hard to produce. Therefore, for Nb24-containing constructs we preferred to express only a few hours instead of overnight, and during purification, Tris buffers with elevated salt concentrations (150 mM) seem to give higher yields instead of standard PBS. The production and purification of recombinant DR5 and TRAIL was even more tricky. During SEC of DR5 we noticed the presence of oligomer structures, possibly due to free sulfhydryl groups of the CRD in the bacterial cytoplasm of Shuffle cells that start to oxidize and form intermolecular disulfide bonds. We therefore, used solutions of lower pH (acetate pH 5.0, to reduce the deprotonation of the sulphydryl group) in our buffers. These minor adaptations in protocol resulted in higher production yields, and single symmetrical elution peaks from SEC. The preparation of TRAIL faced serious aggregation problems. It is well established that TRAIL degrades fast and thus has a fast turn-over. So, it is considered to be a very fragile protein. To stabilize our recombinant TRAIL, we used additives including 10% glycerol, 0.01% mannitol, 8% trehalose, 20 µM zinc chloride and 1 mM dithiotreitol.

According to current models, it seems that the trimeric TRAIL protein interacts with up to three DR5 molecules to form a network on the membrane that triggers apoptosis [6]. To mimic this oligovalency of TRAIL, we generated biparatopic, bivalent, trivalent and tetravalent Nbs with Nb28. The (Gly_4_Ser)_3_ linkers were chosen to separate the individual Nbs within one molecule, since such an artificial spacer is supposedly non-structured, very flexible and it could separate the individual Nbs by up to 50 Å [23]. The bivalent Nb and biparatopic Nb construct is capable of binding two molecules within the dimeric DR5, immobilized on the SPR sensor chip, as evidenced from the Rmax values (Table 1). At this stage, it is not known whether the dimeric DR5 structures in our preparations have the same structure as PLADs, but for sure the DR5 dimer in our preparations comprises two epitopes for Nb24 and also for Nb28. Furthermore, an increased apparent affinity was noticed in SPR for bivalent and biparatopic DR5-specific Nb constructs, which indicates that both entities within the Nb construct were active in antigen binding. The apparent affinity did not increase much for the trimeric Nbs (Table 1). A bispecific Nb construct whereby the DR5-specific Nb is joined with a serum albumin binding Nb (Nb24-A or Nb24-A) exhibited exactly the same binding parameters to DR5 as the monomeric Nb. Thus, we conclude that the addition of an anti-serum albumin Nb to our constructs will be neutral for DR5 binding, but it might represent a good strategy to increase the blood retention of the Nb construct in future therapeutics. 

Our recombinant trimeric TRAIL protein was potent in tumor cell killing, confirming its “native-like” folding. Not all cancer cell lines react in the same way to the recombinant TRAIL, which probably reflects the variable abundancy of DR5 on the different cancer cells. Remarkably, the monomeric Nb28 and even the dimeric Nb28 or biparatopic Nbs failed to induce tumor cell–killing activity. We therefore hypothesize that both entities of the bivalent Nb28_2_ will bind to both DR5 molecules within the dimeric PLAD, without destruction of the PLAD conformation and thus without provoking cytoplasmic signaling and killing. In sharp contrast, the Nb28_3_, Nb28_4_ exhibited strong tumor killing capacity in a dose dependent manner and often as good as TRAIL. This suggests that the association of the trivalent or tetravalent Nb28 construct with the cell surface exposed DR5 manages to trigger the clustering of its antigen and to overcome the auto-inactivated state of the PLAD. The cell toxicity of Nb28_3_ and Nb28_4_ was noticed for Colo205 and PC3 cells but not for HeLa, pointing toward the importance of an elevated DR5 exposure on the cell membrane. 

Unfortunately, as mentioned earlier, the expression of Nb28_4_ was seriously impeded, and only small amounts could be purified. We therefore explored the generation of a bispecific Nb construct, Nb28-113, where the C-terminal Nb would recognize each single B domain of a pentameric Stx2aB_5_ compound. Hence, mixing the Nb28-113 and Stx2aB_5_ will spontaneously generate pentameric Nb28 constructs. Indeed, this mixture was also highly potent in tumor cell killing. 

Our data show that the efficacy of TRAIL is highly dependent on the cancer cell line. It even seems that the number of DR5 molecules on the cell surface is not the only determinant for apoptosis. The affinity, the mechanism of interaction and signal transduction might also affect the apoptosis, autophagy or even survival pathways. In addition, TRAIL is capable of binding to five different types of receptors, where DR4 and DR5 are triggering apoptosis and the decoy receptors (DcRs) are surviving receptors. Therefore, the final outcome will depend on the balance between these engaged, different receptors. Our Nbs are supposedly more specific for DR5 alone and less disturbed by presence of DR4 or DcRs than TRAIL (although it is not yet formally demonstrated). 

Finally, TRAIL binding to DR5 is expected to induce the apoptotic pathway, which should be witnessed by the activation of caspase 3/7. Indeed, the appearance of caspase 3/7 activity correlates well with the killing activity of TRAIL and of the trimeric, tetrameric or pentameric Nb constructs. We therefore, conclude that the presence of these oligomeric Nb28 constructs binds to the cell surface exposed DR5 molecules and induces cytoplasmic signaling that activates the caspase 3/7 and initiates the apoptotic pathway. 

## 4. Materials & Methods

### 4.1. Expression and Purification of DR5, TRAIL, and Stx-2aB_5_

Freshly transformed *E. coli* SHuffle^®^ T7 Express harboring the recombinant plasmids pET-28a/dr5 (encoding amino acids 56–211) or pET-28a/trail (encoding amino acids 114–281) were inoculated in 5 mL LB broth containing 50 μg/mL kanamycin and cultivated overnight at 30 °C in an orbital shaker at 200 rpm. Then, 1 mL of this pre-culture was used to inoculate 100 mL freshly made TB and grown at 30 °C. The expression was induced with 1 mM IPTG when the culture reached an OD_600nm_ between 0.7–1.0, and incubation was continued for 6 h at 30 °C while shaking. 

The cultures were cooled on ice for 5 min and centrifuged at 8000 g for 15 min at 4 °C. After removal of the supernatant, cell pellets were either stored at −20 °C or subjected to further purification. Cell pellets were resuspended in lysis buffer (300 mM NaCl, 10 mM imidazole, 50 mM Tris-HCl, pH 8.0) and sonicated for 10 min (in intervals of 10 s). Samples were then centrifuged at 8000 g for 35 min to remove cell debris. The soluble proteins were applied on HisPur Ni-NTA resin (Thermo Scientific, Waltham, MA, USA), washed extensively with lysis buffer including 30 mM extra imidazole. and recombinant protein was eluted in buffer containing 150 mM imidazole in six separate fractions of 1–2 mL. The fractions containing recombinant protein were pooled and applied on Sephadex-75 column equilibrated in PBS for TRAIL preparation and in 10 mM acetate pH 5.0 for DR5 preparation.

The B subunit of Shiga-like toxin 2a was produced recombinantly in *E. coli* BL21. This protein B domain forms spontaneously a homopentamer when expressed and purified as described earlier [14].

### 4.2. Expression and Purification of DR5-Binding Nbs

DR5-binding Nbs were identified after immunizing a five-year-old dromedary with soluble DR5 protein, construction of an immune Nb library from blood and lymph nodes in pComb3X vector, and enrichment by phage display and panning following published methods [20]. After choosing the best DR5-responsive clones in ELISA the chimeric pComb3X phagemid was extracted from TG1 *E. coli* cells. Its Nb sequence was ligated into pMECS or pHEN6c vectors between PstI and BstEII (pMECS) or EcoR I (pHEN6c) restriction enzyme sites and transformed in *E. coli* WK6 cells. The transformed cells were plated on LB agar plates with 2% glucose and 100 µg/mL ampicillin and incubated overnight at 37 °C. After verifying the constructs by nucleotide sequence analysis, a single colony from the plates was cultured in TB medium supplemented with 0.1% (w/v) glucose, 100 μg/mL ampicillin, and 2 mM MgCl_2_. The Nb expression was induced with 1 mM IPTG when the culture reached an O.D._600nm_ between 0.6–0.9, and incubation was continued overnight at 28 °C while shaking. With the pMECS vector the recombinant protein with a C-terminal hemagglutinin tag and His_6_ tag, whereas the recombinant protein expressed from pHEN6 vectors will only carry the His_6_ tag. The periplasmic proteins were extracted by osmotic shock and purification of His-tagged Nbs was performed as described before [14]. The concentration of purified Nbs was measured via UV spectrophotometry on NanoDrop 2000 (Thermo Scientific) using the extinction coefficient predicted with the ExPASy ProtParam tool.

Purity of Nbs was assessed by SDS-PAGE staining with Coomassie blue. A western blot using anti-His Tag monoclonal antibody (Biolegend, San Diego, CA, USA) and goat anti-mouse IgG conjugated to horse radish peroxidase (Sigma Aldrich, St Louis, MO, USA) was used to confirm the identity of the Nb. 

The expression of bi, tri, and tetravalent monospecific Nbs and the bispecific Nbs was performed exactly as explained for the monomeric Nb. 

### 4.3. Cloning of Bivalent, Trivalent, or Tetravalent Monospecific Nb Constructs

The plasmid encoding, what will be, the second Nb was digested with PstI and NcoI restriction enzymes, while the gene fragment containing the first Nb sequence was amplified by PCR with primers encoding a (Gly_4_Ser)_3_ linker. The amplicon was also digested with the same nucleases. During PCR, a (Gly_4_Ser)_3_ encoding linker containing a unique PstI site was added downstream of the Nb template, whereas the primer annealing at the 5′ end of the amplified Nb gene enforced a NcoI restriction enzyme site and removed the PstI site within the framework-1 region of the Nb. The digested products were cleaned and finally ligated using T4 DNA Ligase (Thermo Fisher Scientific). The ligation product was transformed in *E. coli* WK6 competent cells, plated on LB agar containing glucose (2%) and ampicillin (100 µg/mL), and incubated overnight at 37 °C. A few colonies were picked individually and PCR was performed to confirm the presence of an insert with a size corresponding to the tandem Nb genes separated by the (Gly_4_Ser)_3_ linker. The PCR-positive colonies were cultured in LB medium, and their plasmids extracted and sent for sequencing to confirm the presence of the correct insert.

Likewise, for the trivalent and tetravalent constructs, PCR amplified bivalent Nb or trivalent Nb fragments were digested with NcoI and Pst1 and applied on agarose gel. The band corresponding to the bivalent or trivalent Nb was purified from agarose gel and ligated in the pMECS vector containing a Nb cut with the same nucleases to arrive at the trivalent and tetravalent, monospecific Nb constructs, respectively. 

### 4.4. Cloning of Bispecific Nb Constructs

We also replaced one of the DR5-specific Nbs with the Nb SA1 directed against serum albumin of mice and humans or with the Nb113 against the B subunit of Shiga toxin 2a (Stx-2aB_5_). In this case, a monovalent or bivalent DR5-Nb construct was amplified by PCR, cut by NcoI and PstI, and purified on agarose gel, before ligation in the pHEN6c vector containing the NbSA1 or Nb113, respectively. A (Gly_4_Ser)_3_ linker was spacing always the different Nbs. All constructs were confirmed by nucleotide sequencing. 

### 4.5. Western Blot Using Nbs as Probe

DR5 protein (5 µg per lane) was separated by SDS-PAGE under reducing and non-reducing conditions. The proteins were transferred onto Protran 0.45 µm NC nitrocellulose western blotting membrane (Amersham, GE Healthcare, Buckinghamshire, UK) and residual protein binding sites on the membrane were blocked by overnight incubation at 4 °C in 2% skim milk in PBS. The membrane was soaked for 1 h at room temperature (RT) in purified Nb (25 µg/mL). The membrane was washed three times with PBS, and mouse anti-HA tag monoclonal antibody (Biolegend)(1:2000 in blocking buffer) was added and incubated for 1 hr at room temperature to bind to the hemagglutinin-containing Nb. Subsequently, the membrane was washed three times with PBS, and goat anti-mouse IgG HRP-conjugated (Sigma Aldrich) was added (1:2000 in blocking buffer) and incubated for 1 hr at room temperature. Finally, the membrane was washed three times with PBS before adding 18 mg of 4-chloro 1-naphtol in 6 mL ethanol, 30 mL buffer (500 mM NaCl, 25 mM Tris pH 7.5) and 18 µL H_2_O_2_ for colorimetric visualization of the DR5 bands.

### 4.6. Affinity Measurement and Epitope Binning

To determine the binding properties of Nbs to DR5, an SPR experiment was performed on Biacore T200 (GE Healthcare) according to the manufacturer’s instructions. The CM5 sensor chip (GE Healthcare), was used for capturing DR5 at a concentration of about 5 μg/mL and a flow rate of 10 μL/min for 1 min. This resulted in coupling of ~150 RU DR5 to the sensor chip surface. Meanwhile, one flow cell of the sensor chip was left without captured DR5 to provide a reference surface. Nanobodies were prepared at different concentrations starting from 125 nM or 10 nM using a 2-fold serial dilution in running buffer (150 mM NaCl, 0.005% Tween-20, 3.4 mM EDTA, 20 mM HEPES, pH 7.4). 

For each Nb construct, sensorgrams were recorded for different analyte concentrations at a flow rate of 30 µL/min and a data collection rate of 1 Hz. Analyte injections were performed with association and dissociation phases of 180 s and 300 s, respectively. Prior to data analysis, reference and zero concentration data were subtracted from the sensorgrams. The collected data were fitted according to a 1:1 Langmuir binding model (one Nb to one monomer of DR5) from the experimental flow cells of a single biosensor chip. All experimental data were treated with Biacore T200 Evaluation Software to calculate the kinetic k_on_ and k_off_ rates and the equilibrium dissociation constant (K_D_).

For epitope binning, we first injected an excess of Nb-A (a concentration equivalent to 100 times its K_D_ value) for 300 s to saturate all epitopes on the DR5. This was followed by applying a mixture of excess Nb-A and Nb-B (both at a concentration of 100 times their K_D_ value) and monitoring the resonance units (RU) over a period of 300 s. The chip was then injected with buffer without any Nb for 500 s. All possible Nb combinations were tested including all orders of injection. 

### 4.7. Flow Cytometry

The Hela cell line was obtained from dr C. Goyvaerts (VUB, Jette, Belgium). The Colo205 cell line was provided by dr C. Vangestel from UZA Edegem, Belgium. The HeLa, PC3, and LLC1 cell lines were originally obtained from American Type Culture Collection (ATCC) (Manassas, VA, USA).

HeLa (ATCC CCL-2, Colo205 (ATCC CCL-222), PC3 (ATCC CRL-1435), and LLC1 ATC CRL-1642) cells were cultured according to the specifications of the ATCC. The (semi-)adherent PC3, HeLa, and Colo205 cells were collected after trypsinization (ThermoFisher Scientific, Gibco, #25200072) and washed twice with HBSS Buffer (Gibco #14025050). DR5 specific Nbs (10 µ/mL) were mixed with 100 µL cell suspension (10^6^ cells/mL). After incubation for 1 hr, cells were washed with ice-cold HBSS, resuspended with HBSS Buffer, and then APC labelled anti-human CD262 (DR5, TRIAL-R2) antibody (Biolegend) or one of our Nb constructs plus APC labelled anti-His monoclonal antibody (Biolegend) were added. Incubations where performed on ice and washings were at 4 °C, and cells were analyzed on FACS Canto II (BD Biosciences, Erembodegem, Belgium).

### 4.8. Cell Survival Assay

Cells were plated in a clear-bottom 96-well plate (Sigma Aldrich, Costar, #3903) at 5000 to 10,000 cells/well in dedicated media. The next day, serially diluted Nbs or Apo2L/TRAIL was added to the wells. After 48 h, cell viability was measured using AlamarBlue^®^ (Bio-Rad, # BUF012A, Hercules, CA, USA), and absorbance of the solution in the wells was determined with a standard microplate ELISA reader.

### 4.9. Caspase Activity

Cells were plated at 5000 to 10,000 cells/well and left to attach overnight at 37 °C. The next day, the 50 µL culture in the wells was supplemented with 50 µL media containing the indicated concentrations of DR5 agonists. Caspase activity was measured using CaspaseGlo-3/7 kit (Promega, #G8202, Madison, WI, USA) according to the suppliers’ instructions. 

## Figures and Tables

**Table 1:** Kinetic parameters of Nb–DR5 interactions measured by surface plasmon resonance (SPR).

Nb	k_on_ (M^−1^s^−1^)	k_off_ (s^−1^)	KD (M)	χ^2^	R_max_ (RU)	Expression Yield
Nb 24	1.83 × 10^5^	1.31 × 10^−3^	7.17 × 10^−9^	1.05	106	1.2 mg/L
Nb 28	3.14 × 10^6^	6.88 × 10^−4^	2.19 × 10^−10^	0.17	117	2.6 mg/L
Nb 28_2_	4.57 × 10^6^	7.58 × 10^−5^	1.66 × 10^−11^	0.391	145	2.2 mg/L
Nb 28_3_	3.49 × 10^6^	6.98 × 10^−5^	2.00 × 10^−11^	1.13	154	0.13 mg/L
TRAIL	6.91 × 10^5^	3.13 × 10^−4^	4.53 × 10^−10^	45.8	300	1.6 mg/L
Nb 24-A	1.16 × 10^5^	1.13 × 10^−3^	9.79 × 10^−9^	1.36	209	0.5 mg/L
Nb 28-A	1.72 × 10^6^	6.82 × 10^−4^	3.96 × 10^−10^	0.0823	234	0.2 mg/L
Nb 28-24	1.39 × 10^6^	3.80 × 10^−5^	2.73 × 10^−11^	0.0821	180	0.15 mg/L
Nb 24-28	1.55 × 10^6^	8.48 × 10^−5^	5.47 × 10^−11^	0.337	187	0.12 mg/L
